# Book Reviews

**Published:** 1902-08

**Authors:** 


Diseases of the Nose, Pharynx and Ear. By Henry Gradle, M. D. Professor of Ophthalmology and Otology, Northwestern University Medical School, Chicago. Octavo of 547 pages, profusely illustrated, including two full-page plates in colors. Philadelphia and London: W. B. Saunders & Co. 1902. [Cloth, $3.50 net.]

The rapid strides made in the topics of medicine treated of in this book within the last few years, toward perfected principles and practice, have been pointed to over and over again. This work further emphasises the fact in an incisive way. The author has taught in these branches for a quarter of a century and presents his personal experiences in a clear and concise fashion.

The anatomy of the naso-pharynx is admirably depicted in text and strongly illustrated in engravings. No better pictures of the upper air tract have ever been published in a textbook. In handling diseases of this region the author has presented many of the questions in an original manner, and gives information not usually accessible.

The work is divided into two parts, or books as they are termed, the first being devoted to the consideration of diseases of the nasal passages and pharynx; and the second is set apart to discussion of diseases of the ear. The methods of examination, with all the various instruments of value are described and the author's technic is to be commended. Diseases of the sinuses, especially the maxillary, are given appropriate space and are treated with the most modern scientific methods.

We are specially attracted to the chapter on retronasal catarrh, because the affection is so common and is very inadequately described or detailed in the usual textbooks. This author gives it due importance, but he confesses that there is no known treatment that can effect its cure. It is to be hoped that this will not long be so.

Infectious diseases of the upper air tract,—diphtheria, syphilis, tuberculosis,—are set forth most interestingly as well as scientifically and their exposition bespeaks a thorough acquaintance with their clinical manifestations and with their essential management. Tumors of the nose and pharynx are also well handled, and appropriate operations are advised and described. The septum and turbinals, the special field of the amateur, are given, in their description here and there is a proper pathological place and their importance in a rhinological sense is duly considered.

The second book takes up the anatomy and physiology of the ear, discusses the etiology of ear disease, and then proceeds to describe symptoms and treatment, chapter by chapter, until a most complete monograph of the subject is written. The general physician should read the chapters relating to diseases of the middle-ear, because they are fresh, recent, and will aid him in the important early detection of these affections. The whole subject of ear disease is dealt with masterfully and is a credit to the author as well as a real contribution to literature. Finally, the entire treatise contains so much to commend and so little to criticise, that we yield to it our unqualified endorsement as one of the best of the recent works on these diseases.

A SYSTEM OF PHYSIOLOGICAL THERAPEUTICS. A Practical Exposition of the methods, other than drug-giving, useful in the prevention of Disease and in the Treatment of the Sick. Edited by SOLOMON SOLIS COHEN, A. M., M. D., Professor of Medicine and Therapeutics in the Philadelphia Polyclinic; Lecturer on Clinical Medicine at Jefferson Medical College; Physician to the Philadelphia Hospital and to the Rush Hospital for Consumption, etc. In eleven 8vo volumes. American, English, German and French authors. Volume IX. Hydrotherapy, Thermotherapy, Heliotherapy and Phototherapy. By Dr. Wilhelm Winternitz, Professor of Clinical Medicine in the University of Vienna; Director of the General Polyclinic in Vienna; Assisted by Dr. Alois Strasser, Instructor in Clinical Medicine at the University of Vienna, and Dr. B. Buxbaum, Chief Physician of the Hydrotherapeutic Institute in Vienna. And Balneology and Crounotherapy by Dr. E. Heinrich Kisch, Professor in the University of Prague; Physician at Marienbad, Spa. Translated by Augustus A. Eshner, M. D., Professor of Clinical Medicine in the Philadelphia Polyclinic, etc. Illustrated. Philadelphia: P. Blakiston's Son & Co. 1902. [Price for the set complete, $27.50 net.]

A scientific treatise upon the subjects embraced in this volume has been needed for some time past. There has been enough literature afloat, but it has partaken for the most part of the nature of commercialism. Now, we have something that can be relied upon as a substantial guide in the management of the worn and invalided, — the neurasthenic and exhausted,—who come to us for advice rather than medicine. At any rate these groups of patients need, pretty generally, the kind of treatment set forth in this book, more than that which is obtained at the drug shop.

It seems remarkable that such valuable therapeutic means as are described in this work should be left so largely to the empiric, and that patients who most need these means can usually find them in "institutions" or sanatoria managed by so-called physicians who, to say the least, have no claim to the title in a scientific sense, and who are interested solely in the size of the bank accounts of their customers. The time has come for a change, and educated physicians who are trained in the best science the period affords, must be encouraged to take the management of infirmaries where all the various methods of treatment set forth in this book can be obtained at reasonable price and applied under a scientific eye.

We trust this work will find its way into the hands of every physician who possibly may be called upon to prescribe for those who need these methods of treatment.

---

A Handbook of Appendicitis. By A. J. Ochsner, M. D., Professor of Clinical Surgery, College of Physicians and Surgeons, Medical Department of the University of Illinois; Surgeon of the Augustana Hospital, etc. Pp. 182. Illustrated. Chicago; G. P. Englehard & Co. 1902. [Price, $1.00 net.]

This well written and handsomely printed monograph is a valuable addition to the literature of appendicitis. It embraces the important features of the subject from anatomy to post-operative treatment,—everything essential to the masterful conduct of the disease.

In ten lucid chapters of exceptionally clear, pure and simple English, Ochsner tells the story of a disease that is of constantly increasing importance, and though much has been written upon it, the last word has by no means been said. It should be studied in all its aspects by every general practitioner of medicine, that he may know how to act in the sudden presence of the malady, and this brochure of Ochsner will help him vastly in making up his judgment. Its size is convenient for the pocket, hence may be made a constant companion. A bibliography and index, both of great moment, close a book that is deserving of the highest praise.

The Practical Medicine Series of Year Books. Ten volumes. Issued monthly under the general editorial charge of Gustavus P. Head, M. D., Professor of Laryngology and Rhinology in the Chicago Post-Graduate Medical School. Volume IV. Gynecology. Edited by Emilius C. Dudley, A. M., M. D., Professor of Gynecology, Northwestern University Medical School; Gynecologist to St. Luke's and Wesley Hospitals, Chicago. With the Collaboration of William Healy, A. B., M. D. 12mo, pp. 212. Illustrated. Chicago: The Year Book Publishers. 1902. [Price, $1.25.]

The editor of this volume, Dr. Dudley, has become one of the most advanced, painstaking and skilful of American gynecologists. It would be an impossibility to attach his name in any way to a gynecological treatise that did not possess merit.

We regard this number of the Practical Medicine Series as one of the most valuable yet issued. The material has been carefully selected with reference to exhibiting the most noteworthy contributions to gynecological literature made during the year 1901. The book is of special value to the advanced student and junior physician, though it is full of interest to the more experienced gynecologist,—even the specialist may derive much benefit from its careful study. Some of the illustrations are exceptionally fine, while all are well chosen and afford material aid to the study of the text.

---

A Practical Treatise on Smallpox. Illustrated by colored photographs from life. By George Henry Fox, M.D., Consulting Dermatologist to the Health Department of New York City. With the Collaboration of S. D. Hubbard, M. D., S. Pollitzer, M. D. and J. H. Huddleston, M. D. In two parts, atlas form. Philadelphia and London: J. B. Lippincott Co. 1902. [Price, $3.00.]

The author of this work most truthfully remarks that when a physician is called to a case of suspected smallpox, he confronts a grave responsibility. A mistake made here may cost lives and treasure—to say nothing of reputation, which once lost is not easily regained.

The object of this treatise is to equip the physician with just the information needed to avoid such direful mistakes as are made all too frequently in the differential diagnosis of the early stages of smallpox. It is at once practical and elaborate—practical in its simplicity and conciseness of its text, elaborate in its illustrations, many of which have never been equaled and none excelled. It is, the author believes, the first presentation of the eruption in each of its successive stages, the plates in color tints being reproduced from life. Dr. Fox is experienced with the camera and his collection of photographs, showing all manner of skin diseases, is famous among dermatologists. Those used in this work are rare specimens, obtained sometimes under stress, and present the disease in its various stages, the day of the eruption being given in the legends, and altogether makes the most complete exposition of smallpox yet attempted. It is a timely contribution to the subject and ought to do good service in preventing the spread of a loathsome disease that should not be permitted to exist in these enlightened days when sure prevention is at hand.

---

THE DIAGNOSIS OF SURGICAL DISEASES. By DR. E. ALBERT, late director and Professor of the first Surgical Clinic at the University of Vienna. Authorised translation from the eighth enlarged and revised edition, by Robert T. Frank, A. M., M. D. Octavo, pp. 427. With 53 illustrations. New York: D. Appleton & Co. 1902. [Price, cloth, $5.00.]

This work appears for the first time in English, though it long has been popular with those who read it in the native tongue of the author. It considers a subject of great moment to every surgeon, or those who practice surgery even occasionally, and it takes conditions up as they are met with clinically or at the bedside. Albert has had the supreme advantage of obtaining his material from the great clinics of Vienna, where there is practically no limit to it, and where surgery is seen at its best.

A treatise on medical diagnosis is no novelty, but one exclusively devoted to the diagnosis of surgical diseases and injuries is unique. The language is clearly expressive of the author's meaning, and there has been no attempt to discuss theories; but the several diseases possessing similarity in symptomatology are grouped and their similars described and differentiated.

Many of the clinical evidences of disease are graphically described and a large number of cases are presented and analysed. Here is an example:

> An old gentleman fractured his thigh while out walking, but not at the neck of the femur as is usually the case with old people. When v. Dumreicher was called in, he noticed a strabismus of minor degree. He immediately followed up the symptom, and on questioning found the strasbismus had existed fourteen days. He diagnosticated fracture due to carcinomatous degeneration of the bone, and also carcinoma at the base of the skull. Autopsy confirmed the diagnosis. The primary focus was a carcinoma of the parotid.

In a chapter devoted to the consideration of thoracic tumors the author describes the differentiation of those having surgical importance in a manner especially interesting, and likewise in a subsequent chapter handles abdominal tumors in a similarly instructive and engaging fashion. As might be anticipated in such a work there is little attempt at illustration, though a few drawings are introduced that serve a good purpose. We expect to see, some day, an American treatise of similar character profusely illustrated. Meanwhile, there is no better work than this upon a similar topic, and it will prove of great aid to every physician in the country who must needs practise surgery.

---

International Clinics. A Quarterly of Clinical Lectures and Especially Prepared Articles on Medicine, Neurology, Surgery, Therapeutics, Obstetrics, Pediatrics, Pathology, Dermatology, Diseases of the Eye, Ear, Nose and Throat, etc. Edited by Henry W. Cattell, A. M., M. D., of Philadelphia, with the collaboration of John B. Murphy, M. D., of Chicago: Alexander D. Blackader, M. D., of Montreal; H. C. Wood, M. D., of Philadelphia; T. M. Rotch, M. D., of Boston; E Landolt, M. D., of Paris; Thomas G. Morton, M. D., of Philadelphia; J. W. Ballantyne, M. D., of Edinburg, and John Harold, M. D., of London. Volume I. Twelfth series. 1902. 84 illustrations, with 3 colored plates. Philadelphia: J B. Lippincott Co. [Price. cloth, $2.00 each; half-leather, $2.25 each.]

The excellent standard established in the beginning of the publication of these clinics is not only still maintained, but is improved upon year by year and number by number. The present volume is particularly noticeable in the variety and excellence of its subjects and in the number and quality of its illustrations. The contents include lectures on therapeutics, medicine, surgery, obstetrics, and diseases of the ear; also two biographical sketches of living medical men,—S. Wier Mitchell and John A. Wyeth,—and a final section called progress of medicine. This latter is a review of the literature of note during the year 1901.

The book, as a whole, cannot fail to interest the practising physician if he would keep pace with the events in his profession that are of importance.

---

Manual of Childbed Nursing, with Notes on Infant Feeding. By Charles Jewett, A. M., M. D., Sc. D., Professor of Obstetrics and Diseases of Women in the Long Island College Hospital. 12mo, pp. 84. Fifth edition, revised and enlarged. New York: E. B. Treat & Co. 1902. [Price 80 cents.]

This excellent manual is one of the clearest, most concise and best of the several brochures pertaining to nursing in the lying-in chamber. It contains scarcely a superfluous word, yet everything of importance pertaining to childbed nursing can be found between its covers. The author is one of the foremost teachers and his experience has been practically applied in the making of this book. It should be placed in the hands of every mother, and every obstetric nurse needs it, as a matter of course.

---

PROGRESSIVE MEDICINE, Vol. II., June, 1902. A Quarterly Digest of Advances, Discoveries and Improvements in the Medical and Surgical Sciences. Edited by HOBART AMORY HARE, M. D., Professor of Therapeutics and Materia Medica in the Jefferson Medical College of Philadelphia. Octavo, 440 pages, 28 illustrations. Philadelphia and New York: Lea Brothers & Co. [Per annum, in four cloth-bound volumes, $10.00. Per volume, $2.50, by express prepaid to any address.]

The subjects dealt with in this volume are surgery of the abdomen, including hernia, by William B. Coley; gynecology, by John G. Clark; diseases of the blood and ductless glands, the hemorrhagic diseases, and the metabolic diseases, by Alfred Stengel; and ophthalmology, by Edward Jackson.

The surgery of the abdominal viscera is espcially interesting, containing as it does the later methods of dealing with the kidney, liver and pancreas. The gynecological field, too, has been well covered, and in particular has cancer of the uterus received some attention. The great importance of this topic is being impressed upon the profession in every recent publication relating to it, and in the brief consideration given it here, we find reflected the most advanced views.

Diseases of the blood, as might be expected, are most ably and thoroughly discoursed upon and many useful hints in pathology and diagnosis are advanced. The section on ophthalmology will prove of interest to specialist and generalist. The entire volume is a condensation of progress that deserves and ought to receive approbation.

